# Tobacco Smoking and Cardiovascular Health in Africa: Trends, Interventions, and Policy Implications—A Narrative Review

**DOI:** 10.1002/hsr2.73297

**Published:** 2026-09-23

**Authors:** Boluwatife Samuel Fatokun, Success Oluwapemi Oluyemi, Obinna Joseph Mba, Omosola Lydia Bolarin, Ugochi Chinonyerem Ajaero, Ahmed Muhammad Babandi, Chukwuebuka Udo Onyegiri, Pascal Mathew Okorobe

**Affiliations:** ^1^ Faculty of Basic Medical Sciences Kwara State University Malete Nigeria; ^2^ Department of Nursing Science Osun State University Osogbo Nigeria; ^3^ Department of Pharmacology and Toxicology David Umahi Federal University of Health Sciences Uburu Ebonyi Nigeria; ^4^ Department of Biochemistry Southwestern University Nigeria Ogun State Nigeria; ^5^ Faculty of Medicine University of Nigeria Nsukka Nigeria; ^6^ Department of Human Anatomy Federal University Dutsinma Katsina Nigeria; ^7^ Department of Internal Medicine Federal Medical Centre Jabi Abuja Nigeria; ^8^ School of Medicine Makerere University Kampala Uganda

**Keywords:** Africa, cardiovascular disease, public health, tobacco control, tobacco smoking

## Abstract

**Background and Aims:**

Tobacco smoking in Africa presents a significant public health challenge, particularly concerning cardiovascular health among young adults and men. While global smoking prevalence has declined, World Health Organization (WHO) projections estimate that the total number of tobacco smokers in Africa will rise from 57 million in 2015 to 67 million by 2025. This trend compounds the region's existing burden of cardiovascular diseases (CVDs). This narrative review aims to analyze tobacco smoking trends, elucidate the link between smoking and CVDs, evaluate implemented interventions, and assess policy responses across African nations.

**Methods:**

This literature review was performed using electronic search databases comprising PubMed/MEDLINE, Google Scholar, and African Journals Online (AJOL). Using keywords and associated Boolean operators pertaining to “tobacco smoking”, “cardiovascular health”, and “Africa”, studies were selected based on their relevance and content quality. Studies focusing on tobacco use and CVD risks within African regions were included. Relevant references from regional public health and medical databases were also included.

**Results:**

While relative smoking prevalence has experienced marginal declines, the absolute number of tobacco users in Africa is projected to rise significantly. This is due to rapid population growth, demographic shifts, and commercial determinants such as aggressive industry marketing and weak regulatory frameworks. This trend worsens CVDs like heart attacks, strokes, and hypertension. Interventions such as public awareness campaigns, graphic warning labels, and progressive taxation show promise but suffer from inconsistent enforcement.

**Conclusion:**

Addressing these trends requires evidence‐driven policy enforcement and multi‐sectoral collaboration to mitigate tobacco smoking‐related cardiovascular risks. Prioritizing targeted interventions for vulnerable populations remains critical to reducing projected regional health and economic burdens.

**Abbreviations**
ACBF: African capacity building foundation
BAT: British American tobacco
CHD: coronary heart disease
CVDs: cardiovascular diseases
FCTC: framework convention on tobacco control
GSTHR: global state of tobacco harm reduction
HCWs: healthcare workers
ITGA: international tobacco grower's association
LMICs: low and middle‐income countries
MI: myocardial infarction
NRT: nicotine replacement therapy
NTCA: national tobacco control act
SC: smoking cessation
TTCs: transnational tobacco companies
UN: United Nations
WEF: World Economic Forum
WHO: World Health Organization

## 1 Background

Tobacco smoking presents a growing public health concern in Africa [1]. Its prevalence rates have continued to rise among different populations and especially amongst young adults and men [1]. Cultural acceptance and aggressive marketing are just a few of the many factors that have contributed to this increased tobacco use [1]. Cardiovascular diseases (CVDs) are a leading cause of morbidity and mortality in Africa, with tobacco use remaining a key contributor over the years [2]. The WHO recorded that, globally, households and national governments have lost a cumulative of more than $1400 billion in healthcare costs and human productivity to tobacco use [2]. This is consistent with regional economic estimates from the African Capacity Building Foundation (ACBF), which reported that Nigeria and Togo lost an estimated $591 million and $4 million, respectively, to medical treatment and productivity decline associated with tobacco use [2]. Hence, tobacco use is not only increasing the risk for CVDs but also intensifying the already present poverty [2].

Over the past two decades, global tobacco smoking prevalence has seen a marginal decline; however, Africa remains a region of concern [3]. According to WHO regional data, the overall relative smoking prevalence among adults in the African region dropped slightly from 14.2% in 2000 to 10.8% in 2015. However, current projections by the WHO indicate that the number of people smoking tobacco in Africa will rise from 57 million in 2015 to 67 million by 2025, reflecting massive growth in total population and the continent's ongoing public health challenge [3]. This trend underscores the urgency for comprehensive tobacco control measures across African nations [3].

This review aims to analyze trends in tobacco smoking, elucidate the focus on the link between tobacco smoking and cardiovascular diseases, evaluate interventions implemented to curb tobacco use, and assess the policy responses across African countries.

## 2 Methods

This literature review was performed using electronic search databases comprising PubMed/MEDLINE, Google Scholar, and African Journals Online (AJOL). To ensure complete coverage of key data to adequately discuss emerging trends and historical policy developments, no date limit was set for the literature search. The initial literature search was completed on March 10, 2025, and an updated search was done on July 12, 2026. Searches were structured using keywords and associated Boolean operators pertaining to “tobacco smoking”, “cardiovascular health”, “tobacco control”, and “Africa”. Two authors independently screened titles, abstracts, and full texts, selecting literature based on thematic relevance and quality of information. A total of 27 references were analyzed to provide insights into smoking trends, interventions, and policy dynamics across the African continent. The characteristics and distribution of all included sources are summarized in Table 1.

**Table 1:** Characteristics of included sources (*n* = 27).

Category	Included sources (*n*)	References	Scope
Peer‐reviewed primary studies	6	Sreeramareddy et al. (2014); Ramsay et al. (2016); Itanyi et al. (2020); Shariful Islam et al. (2022); Mahoto et al. (2023); Mambo et al. (2024)	Smoking prevalence, cardiovascular risk factors, and barriers to healthcare service delivery.
Multi‐country secondary studies	3	Heydari (2020); Immurana et al. (2021); Theilmann et al. (2022)	Tobacco taxation impacts, product analysis across LMICs, and MPOWER implementation analysis.
Policy analysis and historical reviews	7	Otañez et al. (2009); Mohamed et al. (2018); Oladepo et al. (2018); Udokanma et al. (2021); Akande‐Sholabi & Adebisi (2021); Egbe et al. (2022); Bandara et al. (2025)	Commercial determinants, industry litigation, national policy execution (NTCA, FCTC), and multi‐sectoral tobacco control barriers.
Institutional reports on tobacco control	6	WHO Afro (n.d.); WHO Global (2021); Ethiopia Tobacco Control Policies (2022); WEF (2023); ACBF (2023); GSTHR Senegal (2025)	Economic loss modeling, country case studies, and regional policy monitoring.
Dissertation and published reviews	5	Mlinarić et al. (2020); Adewusi (2021); Peruga et al. (2021); Minja et al. (2022); Bandara et al. (2024)	Cultural influence, gaps in African CVD care, global control achievements, and FCTC implementations.

Abbreviations: ACBF, African capacity building foundation; CVD, cardiovascular disease; FCTC, framework convention on tobacco control; GSTHR, global state of tobacco harm reduction; LMICs, low and middle‐income countries; NTCA, national tobacco control act; WEF, world economic forum; WHO, world health organization.

### 2.1 Inclusion Criteria

Studies were selected based on their specific focus on tobacco use, smoking prevalence, tobacco control policies, and cardiovascular health outcomes within African geographical settings. Selection prioritized peer‐reviewed articles, organizational reports (such as the World Health Organization and African Capacity Building Foundation), and national policy frameworks that provided detailed contextual information on interventions or structural barriers. Only papers written in English were included to maintain data reliability.

### 2.2 Exclusion Criteria

Studies that did not explicitly address tobacco products or focused on unrelated medical or cardiovascular risk factors independent of tobacco use were excluded from this review. Articles that lacked sufficient detail regarding African regional or national settings, or those published outside of peer‐reviewed or verifiable institutional frameworks, were also omitted.

## 3 Results

### 3.1 Trends in Tobacco Smoking and Cardiovascular Health in Africa

Current tobacco use is a key cardiovascular risk factor monitored globally, as recommended by the World Health Organization's (WHO) Global Action Plan on Non‐Communicable Diseases⁠ [4]. An investigation on the association between cigarette smoking and coronary heart disease (CHD) indicated a dose‐response relationship [5]. The correlation showed an increase in risk of developing CHD and myocardial infarction (MI) relative to daily cigarette consumption rate [5]. Smokers who consumed between 1 and 14 cigarettes daily faced a twofold risk of developing MI and CHD, while those who smoked 25 or more cigarettes daily experienced a sixfold risk increase [5]. Using serum cotinine as a biomarker for nicotine exposure, a United States National Health and Nutrition Examination Survey (US NHANES) cohort study demonstrated a relative 25% increase in adjusted CVD risk with each unit increase in cotinine [5]. On the contrary, localized studies within diverse African populations have shown variable dose‐response effects [5].

**Figure 1:**
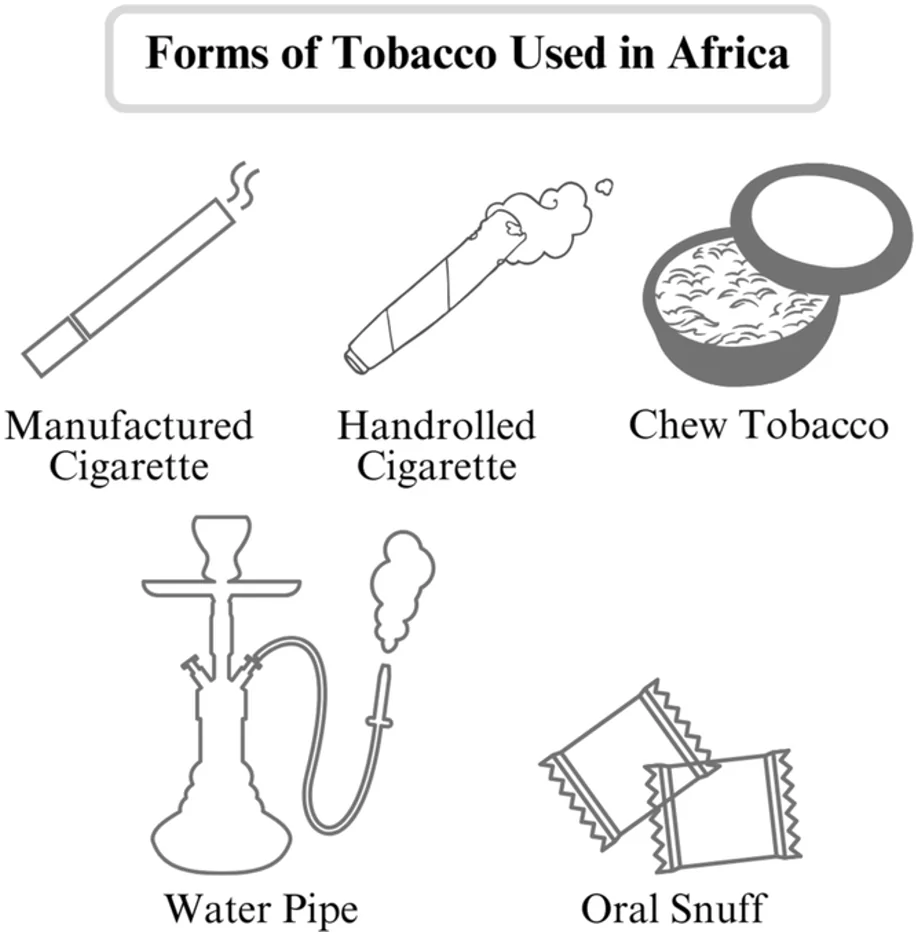
Forms of tobacco used in Africa [4].

A cross‐sectional study of adult survey data across 82 low and middle‐income countries (LMICs) by Theilmann et al. reported a global pooled smoking prevalence of 16.5%, with African adult populations showing wide national variation ranging from 1.1% in Ghana to 20.0% in South Africa and 21.2% in Egypt [4]. Manufactured cigarettes are identified as the most widely used tobacco product in many African countries, alongside hand‐rolled cigarettes, chew tobacco, water pipes, oral snuff, and local variants like “brus” and “cigarillos” as illustrated in Figure 1 [4].

Figure 1 illustrates the primary forms of smoked and smokeless tobacco consumption across the continent, ranging from commercial‐manufactured and hand‐rolled cigarettes and water pipes to chewable tobacco and oral snuff. This highlights the presence of both institutional and active local supply chains involved in tobacco use within the region, all of which drive nicotine exposure and associated cardiovascular risks.

Demographic and epidemiological data across the region consistently show that tobacco use is strongly associated with male gender, older age, lower educational attainment, and lower socioeconomic status [6]. For example, a study in four Sub‐Saharan African countries confirmed that the rate of smoking was significantly higher in men than in women [6]. Furthermore, rural communities consistently report higher levels of tobacco use compared to urban residents [7]. This disparity may stem from lower health literacy among populations with limited education, alongside the use of tobacco as a coping mechanism for socioeconomic stress [7]. For instance, the economic burden of tobacco smoking in Malawi has been directly linked to decreased productivity, the priority purchase of tobacco products over essential family needs, and several mental health implications [2]. Social environments also heavily shape these patterns [8]. A study carried out amongst secondary school students in Enugu State, Nigeria, demonstrated that youth smoking initiation is strongly influenced by parental and peer smoking behaviors [8].

The absolute burden of smoking on the continent is projected to rise rapidly due to population growth and demographic shifts [2]. While the WHO reports a rise in the number of tobacco smokers from 49 million in 2000 to 57 million in 2015 and projects that number to rise to 67 million by 2025, recent institutional reports like the 2023 African Capacity Building Foundation (ACBF) report present even more critical projections [2, 3]. Firstly, ACBF reports an increase in the number of tobacco smokers in the African region between 2000 and 2015 from 52 to 66 million, respectively [2]. Also, ACBF projects that the number of African tobacco smokers would increase to 84 million by 2025 [2]. This represents a projected 61.5% relative increase over two decades, making Africa one of only two regions globally expected to experience an absolute rise in tobacco users during this period [2]. Within Africa, the trend of prevalence levels shows higher rates in Sub‐Saharan African countries like Nigeria and South Africa compared to North African countries [1]. This divide has been strongly associated with cultural norms and the stricter regulatory enforcement in North African countries [1].

### 3.2 Challenges and Barriers to Tobacco Control in Africa

**Figure 2:**
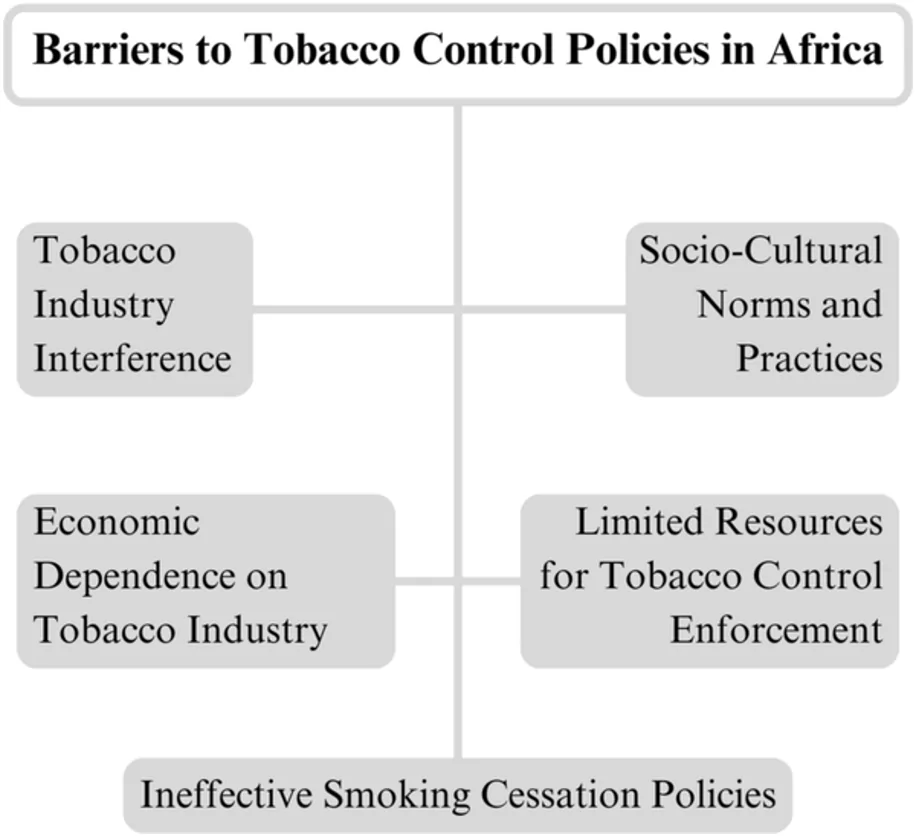
Barriers to tobacco control policies in Africa [2, 9, 12].

Implementing effective tobacco control policies across Africa is hindered by a complex combination of commercial, economic, infrastructural, and socio‐cultural barriers, as shown in Figure 2. A primary challenge is the persistent interference of the tobacco industry in the development and execution of public health policies [9]. Transnational Tobacco Companies (TTCs) actively exploit weak regulatory frameworks to undermine tobacco‐control laws, bypass existing legislation, and drive demand in low and middle‐income nations [9, 10]. These strategies include the introduction of alternative nicotine products, such as oral nicotine pouches (oral snuff), which often evade current regulatory definitions, as well as launching protracted legal challenges [2]. For example, industry‐led litigation in Kenya created a prolonged delay between the ratification of the WHO Framework Convention on Tobacco Control (FCTC) and the implementation of the national Tobacco Control Act [9]. Similarly, in Nigeria, implementation of the National Tobacco Control Act (NTCA) has faced persistent industry resistance, particularly from British American Tobacco [11].

Figure 2 illustrates the multi‐dimensional challenges obstructing the implementation of the World Health Organization Framework Convention on Tobacco Control (FCTC) across the African region. These commercial factors operate alongside systemic health infrastructure deficits, absent cessation resources, and collectivist socio‐cultural practices to weaken the enforcement of active anti‐smoking interventions.

The effect of this interference is partly supported by national economic dependencies on tobacco agriculture. For example, Zimbabwe's primary export commodities are tobacco and gold [13]. Similarly, in Malawi, Transnational Tobacco Companies (TTCs) like British American Tobacco (BAT) and the International Tobacco Grower's Association (ITGA) have actively leveraged the country's economic reliance on tobacco exports to lobby against global control policies [14]. By aggressively promoting the macroeconomic contributions of tobacco, these industries have successfully pressured governments in both Zimbabwe and Malawi to prioritize protecting their primary agricultural export over public health initiatives, thereby severely delaying the domestic adoption of FCTC guidelines [2, 13].

Infrastructural constraints and lack of funding also severely limit clinical and policy enforcement [12]. Smoking cessation (SC) therapies provided by healthcare workers (HCWs) are critical components of tobacco control, yet their implementation in many African nations faces major hurdles [12]. In the Zambezi region of Namibia, a study showed that many smokers do not receive cessation interventions, and healthcare providers lack necessary educational materials on nicotine cessation and harm reduction [12]. Furthermore, some local HCWs are entirely unaware of national tobacco control laws enacted under the FCTC [12]. This problem has also been compounded by a systemic scarcity of smoking cessation products in the regional pharmaceutical market [12].

These aforementioned enforcement challenges are worsened by weak border controls that facilitate a thriving illicit tobacco trade and devalue taxation strategies in many parts of Africa [2]. For instance, the ACBF country‐specific evaluation report indicated that Senegal loses approximately $5.8 million annually in public revenue due to untaxed, smuggled, and counterfeit tobacco products [2]. Consequently, many Sub‐Saharan countries underutilize tobacco excise taxes due to misconceptions regarding economic impacts and a lack of harmonized tax rates across bordering nations [2].

Finally, socio‐cultural dynamics present unique barriers to tobacco control. In highly collectivist societies, such as Nigeria, deep‐seated cultural values regarding respect for elders mean that youth are frequently sent on errands to purchase or even light cigarettes for older family members, inadvertently encouraging early‐onset tobacco use among teenagers and young adults [15].

### 3.3 Interventions Addressing Tobacco Smoking in Africa

Recent multi‐sectoral efforts to raise tobacco awareness include collaborations with schools for youth education and targeted training for healthcare providers [16]. In Nigeria, structured training programs for community pharmacists to deliver counseling and nicotine replacement therapy (NRT) have proven effective in reducing active tobacco use [17]. This clinical approach is supported by the legislative framework under Nigeria's National Tobacco Control Act (NTCA), which mandates graphic health warnings and public education initiatives to curb initiation rates [17, 18].

National fiscal and clinical policies have also demonstrated significant success in South Africa [18]. The integration of smoking cessation counseling directly into primary healthcare facilities resulted in a documented 20% increase in patient quit attempts [18]. Furthermore, South Africa's progressive tobacco taxation strategy successfully reduced smoking prevalence from 32% in 1990 to 16% in 2010, illustrating how targeted economic policies can dramatically reduce population‐level cardiovascular risks [19].

Other African nations have similarly achieved positive outcomes by combining pharmacotherapy with policy enforcement. In Kenya, the WHO country‐specific evaluation report indicated that NRT integration, counter advertising, and public educational programs led to a decline in the percentage of daily tobacco smokers from 6.0% to 2.7% [16]. In Senegal, the strict enforcement of comprehensive bans on tobacco advertising and smoke‐free mandates in public spaces has contributed to the downward trend in smoking prevalence amongst the general population [18]. Additionally, under the FCTC framework, Ethiopia has adopted robust anti‐smoking policies that have translated into notable improvements in regional cardiovascular health outcomes [16, 20].

### 3.4 Policy Implications for Addressing Tobacco‐Related Cardiovascular Health

Mitigating the rising burden of tobacco‐related cardiovascular diseases urgently requires African nations to transition from basic policy formulation to aggressive enforcement. Governments must utilize robust, localized economic data to debunk industry assertions that higher taxes harm national economies [19]. This data‐driven approach is particularly crucial in countries like Nigeria, where enforcement of the National Tobacco Control Act (NTCA) continues to face industry resistance [11]. Strengthening public health policy requires robust legal defense frameworks, funding streams independent of tobacco revenues, and active partnerships with civil society organizations to monitor and expose industry interference [19, 21].

The comprehensive strategies utilized in Uruguay, Australia, and the United Kingdom include high excise taxes, plain packaging, and integrated clinical cessation programs [22]. Similarly, countries in the Eastern Mediterranean Region have achieved marked improvements in tobacco control by strictly implementing the WHO MPOWER package [23]. These global precedents offer a valuable roadmap for domestic policy adaptation and demonstrate the efficacy of multi‐sectoral tobacco control [22, 23]. However, these frameworks cannot be adopted in isolation. African policymakers must tailor them to regional realities [24]. This includes aligning tobacco control with existing national cardiovascular health strategies and primary healthcare systems to ensure sustainability [24].

Also, to ensure sustainable impact, policy execution should follow a phased, multi‐tiered approach. Implementation should begin with high‐impact initiatives in urban centers [24]. This should include strictly enforcing smoke‐free public zones and comprehensive advertising bans before gradually expanding to rural areas where compliance monitoring is more difficult [24]. Governments must also prioritize progressive, simplified excise tax structures and harmonize tax rates across borders to mitigate the illicit tobacco trade [24]. Advocacy must also include targeted social media campaigns, localized mobile quitting applications, and community‐led interventions to drive behavioral change, particularly among highly connected youth populations [24, 25].

### 3.5 Future Directions and Recommendations

Achieving sustainable reductions in tobacco use and cardiovascular disease across Africa requires a strong foundation of empirical research and systematic evaluation. To successfully back and sustain the proposed policy frameworks, future efforts across the continent must prioritize:

- Research aimed at quantifying the direct, country‐specific economic savings of tobacco taxation against national cardiovascular disease (CVD) treatment costs in order to arm policymakers with empirical data [26].
- Evaluation of the efficacy and safety of localized, culturally tailored behavioral therapies and smoking cessation aids within diverse African populations.
- Investigation of the role of digital health technologies such as mobile surveillance apps in monitoring local compliance, evaluating public health campaign reach, and curbing the illicit cross‐border tobacco trade.
- Rigorous independent investigations into past policy failures before launching new campaigns to ensure improvement in design and structural resilience of new campaigns and interventions [27].

## 4 Conclusion

Tobacco smoking remains a significant, preventable risk factor contributing to the shifting cardiovascular disease burden in Africa, particularly among young adults, men, and rural populations. While regional smoking cessation initiatives, targeted taxation, and public awareness campaigns have shown localized utility, their long‐term efficacy depends on sustained legislative commitment and enforcement. Given the projected increases in absolute smoker numbers across the continent, transitioning from broad, generalized control frameworks to data‐driven, culturally tailored strategies is essential. Strengthening multi‐sectoral collaboration among policymakers, healthcare providers, and civil society remains critical to effectively managing smoking prevalence and improving cardiovascular health outcomes across the region.

## Author Contributions

**Boluwatife Samuel Fatokun:** conceptualization, project administration, visualization, writing of original draft, writing and review of final draft. **Success Oluwapemi Oluyemi:** data curation, writing of original draft. **Obinna Joseph Mba:** data curation, writing of original draft. **Omosola Lydia Bolarin:** data curation, writing of original draft. **Ugochi Chinonyerem Ajaero:** data curation, writing of original draft. **Ahmed Muhammad Babandi:** data curation, writing of original draft. **Chukwuebuka Udo Onyegiri:** data curation, writing of original draft. **Pascal Mathew Okorobe:** writing and review of final draft.

## Funding

The authors have nothing to report.

## Conflicts of Interest

The authors declare no conflicts of interest.

## Transparency Statement

The lead author, Boluwatife Samuel Fatokun, affirms that this manuscript is an honest, accurate, and transparent account of the study being reported; that no important aspects of the study have been omitted; and that any discrepancies from the study as planned (and, if relevant, registered) have been explained.

## Data Availability

Data sharing does not apply to this article as no data sets were generated or analyzed during the current study.
